# Minor intraoral salivary gland tumors: a clinical-pathological study

**DOI:** 10.1590/S1679-45082016AO3749

**Published:** 2016

**Authors:** Dmitry José de Santana Sarmento, Maria de Lourdes Silva de Arruda Morais, Antonio de Lisboa Lopes Costa, Éricka Janine Dantas da Silveira

**Affiliations:** 1 Universidade Estadual da Paraíba, Campina Grande, PB, Brazil.; 2 Universidade Federal do Rio Grande do Norte, Natal, RN, Brazil.

**Keywords:** Salivary glands, minor/pathology, Salivary gland diseases, Salivary gland neoplasms/epidemiology

## Abstract

**Objective:**

To evaluate the clinical-pathological profile of patients with minor salivary gland neoplasms.

**Methods:**

A retrospective study of specific cases diagnosed as benign and malignant tumors of the minor salivary glands was performed. The data were collected from medical records of patients seen at a hospital over a period of 15 years. The sample was made up of 37 cases. For the pathological study, slides containing 5μm thick sections stained with hematoxylin and eosin were used. The data were tabulated using descriptive statistics.

**Results:**

Malignant neoplasms represented 70.3% of cases. The mucoepidermoid carcinoma was the most common neoplasm (45.9%), followed by pleomorphic adenoma (24.4%). Most patients were female (70.3%), aged between 71 and 80 years. The palate (67.6%) and the retromolar region (10.8%) were the most affected sites.

**Conclusion:**

Mucoepidermoid carcinoma was the most common tumor in minor salivary glands. These tumors are more common in females aged over 40 years. The palate was the most common affected site.

## RESUMO

**Objetivo:**

Avaliar o perfil clínico-patológico de pacientes com neoplasias de glândula salivar menor.

**Métodos:**

Foi realizado um estudo retrospectivo de casos específicos diagnosticados como neoplasias benignas ou malignas de glândula salivar menor. Os dados foram coletados dos prontuários dos pacientes atendidos em um hospital no período de 15 anos. A amostra final foi de 37 casos. Para o estudo histopatológico, foram usadas lâminas contendo secções com 5μm de espessura, coradas pela técnica de hematoxilina e eosina. Os dados foram tabulados de forma descritiva.

**Resultados:**

As neoplasias malignas representaram 70,3% dos casos. O tipo histológico mais prevalente foi o carcinoma mucoepidermoide (45,9%), seguido do adenoma pleomórfico (24,4%). A maioria dos pacientes era do sexo feminino (70,3%), com idade entre 71 e 80 anos. O palato (67,6%) e a região retromolar (10,8%) foram os sítios mais acometidos.

**Conclusão:**

O carcinoma mucoepidermoide foi o tumor mais comum das glândulas salivares menores. Estes tumores foram mais comuns em mulheres com mais de 40 anos. O palato foi o sítio mais acometido.

## INTRODUCTION

Salivary gland neoplasms are a distinct group of lesions with varying morphology, which present challenges in their diagnosis and treatment.^(1-6)^ Minor salivary gland neoplasms represent less than 25% of intraoral salivary neoplasms. They have distinct characteristics, especially regarding frequency, distribution, and clinical aspects.

Studies that evaluate the epidemiology of minor salivary gland neoplasms are important. These tumors are often malignant, in particular when compared to neoplasms of major salivary glands. In addition, differences between race and geographic location are also observed.^(7-12)^

The mucoepidermoid carcinoma, adenoid cystic carcinoma, and pleomorphic adenoma are the most common tumors of the minor salivary glands. It is estimated that minor salivary gland tumors represent 0.3 to 1.5% of all biopsies in oral pathology laboratories.^(1,13-18)^ Salivary gland tumors can affect patients at any age and affect more females.^(1,12,17,19)^

Neoplasms of the minor salivary glands are a heterogeneous group of tumors. Epidemiological studies are important to understand their frequency and clinical aspects. Investigations in different populations are essential to observe geographic and racial variations of these unusual tumors.^(1,12,17,20)^

## OBJECTIVE

To evaluate the clinical-pathological profile of patients with minor salivary gland neoplasms.

## METHODS

A retrospective study of cases diagnosed as benign and malignant neoplasms of the minor salivary glands was performed. The data were collected from the medical records of patients at *Hospital Dr. Luiz Antônio*, Natal (RN), over a period of 15 years. The study included all cases with histopathological diagnoses of salivary gland neoplasms (benign or malignant) located in the minor salivary glands. Tumors in parotid, submandibular, and sublingual glands were excluded. The sample was made up of 37 cases. This study was approved by the Research Ethics Committee of the *Universidade Federal do Rio Grande do Norte*, protocol number 115/2005.

Data regarding age, sex, anatomic site and size of the lesion, progression of the lesion, symptoms (pain), presence or absence of ulceration (clinical aspect), and regional and/or distant metastasis were collected from medical records. For the histopathological study, slides containing 5μm thick sections stained with hematoxylin and eosin were used. All cases were evaluated by light microscopy and classified according to the criteria proposed by the World Health Organization.^(21)^ Immunostaining was not necessary.

The data were tabulated using the Statistical Package for Social Sciences (SPSS), version 20.0. Data were presented descriptively.

## RESULTS

The final sample was made up of 37 cases in 15 years of evaluation. Malignant neoplasms were more prevalent, and mucoepidermoid carcinoma (45.9%) was the most observed histological type, followed by pleomorphic adenoma (24.4%) and polymorphous low-grade adenocarcinoma (13.5%). Most patients were female (70.2%), with a ratio of 2.3:1 (Table 1).

**Table 1:** Distribution of benign and malignant tumors of the minor salivary glands, according to histological type and sex

Histological type	Sex		Total n (%)

	Female n (%)	Male n (%)	
Benign tumors			
Pleomorphic adenoma	4 (15.5)	5 (45.5)	9 (24.4)
Basal cell adenoma	1 (3.8)	0 (0.0)	1 (2.7)
Canalicular adenoma	1 (3.8)	0 (0.0)	1 (2.7)
Malignant tumors			
Mucoepidermoid carcinoma	12 (46.2)	5 (45.5)	17 (45.9)
Polymorphous low-grade adenocarcinoma	4 (15.5)	1 (9.0)	5 (13.5)
Adenoid cystic carcinoma	2 (7.6)	0 (0.0)	2 (5.4)
Acinar cell carcinoma	1 (3.8)	0 (0.0)	1 (2.7)
Epithelial-myoepithelial carcinoma	1 (3.8)	0 (0.0)	1 (2.7)

Total	26 (100.0)	11 (100.0)	37 (100.0)

**Figure 1:**
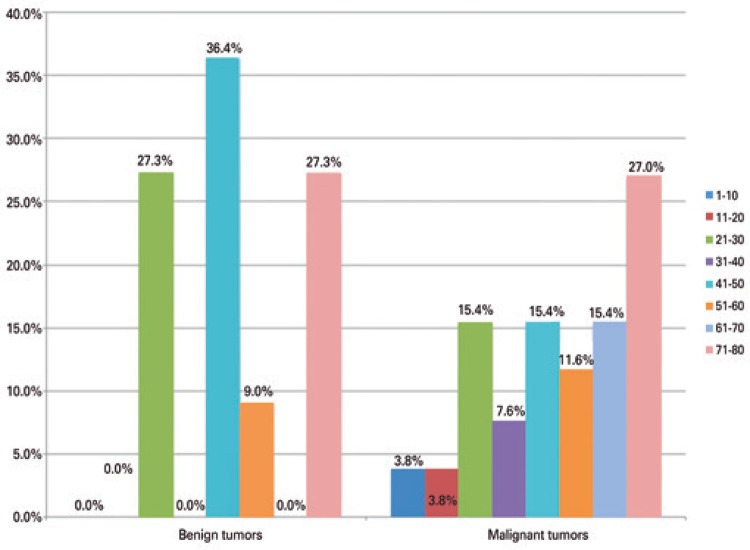
Distribution of benign and malignant tumors of the minor salivary glands per age group

The benign tumors had two peaks in prevalence regarding age: 21 to 30 years and 71 to 80 years. The malignant tumors showed peak prevalence between 71 and 80 years. In general, most patients were aged over 70 years (Figure 1).

In the present study, neoplasms of minor salivary glands were observed mainly in the palate (67.6%) and the retromolar region (15.4%) (Table 2).

**Table 2:** Distribution of benign and malignant tumors of the minor salivary glands, according to histological type and anatomical site

Histological type	Anatomical site						

	Palate	Oral mucosa	Lip	Floor of the mouth	Retromolar area	Tongue	Total
	n (%)	n (%)	n (%)	n (%)	n (%)	n (%)	n (%)
Benign tumors							
Pleomorphic adenoma	9 (100.0)	-	-	-	-	-	9 (100.0)
Basal cell adenoma	-	1 (100.0)	0 (0)	-	-	-	1 (100.0)
Canalicular adenoma	-	-	1 (100)	-	-	-	1 (100.0)
Total	9 (81.8)	1 (9.1)	1 (9.1)	-	-	-	11 (100.0)
Malignant tumors							
Mucoepidermoid carcinoma	9 (53.0)	1 (5.9)	1 (5.9)	1 (5.9)	4 (23.4)	1 (5.9)	17 (100.0)
Polymorphous low-grade adenocarcinoma	4 (80.0)	-	1 (20.0)	-	-	-	5 (100.0)
Adenoid cystic carcinoma	2 (100.0)	-	-	-	-	-	2 (100.0)
Acinic cell carcinoma	-	1 (100.0)	-	-	-	-	1 (100.0)
Epithelial-myoepithelial carcinoma	1 (100.0)	-	-	-	-	-	1 (100.0)
Total	16 (61.6)	2 (7.7)	2 (7.7)	1 (3.8)	4 (15.4)	1 (3.8)	26 (100.0)

Total	25 (67.6)	3 (8.1)	3 (8.1)	1 (2.7)	4 (10.8)	1 (2.7)	37 (100.0)

The progression time of benign tumors was 1.51 year, and the mean of size of lesion was 2.54cm. Only one case (canalicular adenoma) had ulceration, and two patients reported pain. The malignant neoplasms showed a progression time of 2.12 years, and the mean of size of lesions was 2.69cm. Four patients reported pain (three had mucoepidermoid carcinoma) and another four had ulcerations (Table 3). Only one case had metastasized (polymorphous low-grade adenocarcinoma).

**Table 3:** Distribution of benign and malignant tumors of the minor salivary glands per presence of pain and ulceration

Histological type	Pain		Ulceration		Total
	
	Yes	No	Yes	No	
	n (%)	n (%)	n (%)	n (%)	
Benign tumors					
Pleomorphic adenoma	1 (11.1)	8 (88.9)	0 (0)	9 (100.0)	9 (100.0)
Basal cell adenoma	0 (0)	1 (100.0)	0 (0)	1 (100.0)	1 (100.0)
Canalicular adenoma	1 (100.0)	0 (0)	1 (100.0)	0 (0)	1 (100.0)
Total	2 (18.2)	9 (81.8)	1 (9.0)	10 (91.0)	11 (100.0)
Malignant tumors					
Mucoepidermoid carcinoma	3 (17.6)	14 (82.4)	2 (11.7)	15 (88.3)	17 (100.0)
Polymorphous low-grade adenocarcinoma	1 (20.0)	4 (80.0)	2 (40.0)	3 (60.0)	5 (100.0)
Adenoid cystic carcinoma	0 (0)	2 (100.0)	0 (0)	2 (100.0)	2 (100.0)
Acinar cell carcinoma	0 (0)	1 (100.0)	0 (0)	1 (100.0)	1 (100.0)
Epithelial-myoepithelial carcinoma	0 (0)	1 (100.0)	0 (0)	1 (100.0)	1 (100.0)

Total	4 (15.3)	22 (84.7)	4 (15.3)	22 (84.7)	26 (100.0)

## DISCUSSION

Salivary gland tumors are a heterogeneous and rare group of lesions, especially when affecting the minor salivary glands.^(13)^ In the present study, most of the minor salivary gland tumors were malignant, and this data is corroborated by the literature,^(5,11,12,14,17,18,22-28)^ however there are some authors that disagree.^(2,10,13,15,19,29-31)^ The data of the present study are justified by the fact that *Hospital Dr. Luiz Antônio* is a reference center in the care of cancer patients.

Mucoepidermoid carcinoma was the most frequent lesion in this study, followed by pleomorphic adenoma and polymorphous low-grade adenocarcinoma. Our data agree with the studies that consider the pleomorphic adenoma as the most common benign neoplasm of the minor salivary glands.^(5,11,13-15,23,24,26,31)^ Kruse et al.,^(6)^ evaluated only malignant neoplasms of minor salivary glands and observed that adenoid cystic carcinoma was the most prevalent lesion, in disagreement with this study. We suggest that the geographic location of studies and the site where the research was conducted (reference centers for cancer treatment or not) may explain the divergence in these results.^(31)^

In our study, females were more affected by tumors of minor salivary glands, with a ratio of 2.3:1, in accord with other studies.^(5,11,13,15,23,26)^

The mean age was 46.5 years for benign tumors and 51.7 years for the group of malignant tumors, in this study. Malignant tumors appear at a higher mean age when compared to benign tumors. We observed that benign tumors presented two age peaks (21 to 30 and 71 to 80 years) and malignant tumors had an age peak at 71 to 80 years. These results are in agreement with other studies.^(13,14,28)^ However, Jansisyanont et al.^(24)^ reported that malignant tumors can occur in younger patients, in disagreement with the present study. Literature has reported different peaks of age, depending on the histological type analyzed.^(12,13,23,27)^

The palate has been cited as the most common site for minor salivary gland tumors, with prevalence between 42 and 75%. Other anatomical sites involved are the lips (4 to 21%), oral mucosa (5 to 16%), tongue/floor of mouth (4 to 12%), and retromolar area (3 to 7%).^(13-23,24,26,28,30)^ In our study, the most frequent location of tumors, both benign and malignant, was the palate, in agreement with literature data. Oral or labial mucosa was identified as the second most common site.^(14,26)^ These findings differ from the results of the present study that observed the retromolar area as the second most common site.

The floor of mouth, retromolar region, and tongue presented only with malignant neoplasms, in this present study. Venkata et al.,^(26)^ found a statistically significant correlation for minor salivary gland malignant tumors occurring in sites, such as the alveolar mucosa, floor of the mouth, oral mucosa, retromolar area, and intraosseous lesions. Similarly, Pires et al.^(13)^ published that canalicular adenomas were most common in the upper lip, ductal cystadenomas in the lower lip, adenoid cystic carcinomas in the floor of mouth, and acinar cell adenocarcinoma showing a high affinity for the oral mucosa and the upper lip. A recent study reported that adenoid cystic carcinoma of the minor salivary glands is an uncommon tumor with a distinct presentation that occurs in the palate at a late stage (T3-T4), a result with statistical significance.^(32)^

The time of progression was shorter and the mean size of lesions of benign tumors was smaller than for malignant neoplasms. The data were similar between benign and malignant neoplasms, confirming the difficulty in diagnosis of salivary gland tumors. Signs and symptoms can be related to tumor size and may vary according to tumor site. Malignant tumors with a late diagnosis can be related to metastasis, especially adenoid cystic carcinoma.^(1)^

Jansisyanont et al.^(24)^ observed 27.95% (one in four) of malignant tumors present for more than 1 year, and 13.1% (one in seven) were asymptomatic. Therefore, all suspected tumors of minor salivary glands require biopsy to avoid delays and errors in diagnosis. Clinically, it is not possible to differentiate malignant and benign tumors of minor salivary glands.

Swelling was the most common sign of minor salivary gland tumors. Ulceration, ill-fitting dentures, difficulty speaking, and pain were other signs and symptoms observed, and did not significantly differ between benign and malignant tumors. It is not clear if pain is a common sign in malignant neoplasms of minor salivary glands.^(2,4,13,14,20,23)^ Although we observed the presence of pain and ulceration more frequently for malignant tumors in our study, we believe that data are variable in literature and there is insufficient evidence that these characteristics are more common in malignant neoplasms of minor salivary glands.

In this study, no case exhibited lymphadenopathy and only one case showed metastasis. Jaber^(23)^ reported a good survival rate for all histological subtypes of minor salivary gland carcinoma. Jansisyanont et al.^(24)^ reported that only five cases (four high-grade mucoepidermoid carcinomas and one polymorphous low-grade adenocarcinoma) presented with lymph node metastases (8.2%), and two patients died. Histological type, clinical stage, and anatomical site are important in determining prognosis and therapy. Aggressive surgery with wide margins is the best method of treatment for malignant neoplasms of minor salivary gland.^(5,7,11-16,22-24,26)^

## CONCLUSION

Intraoral minor salivary gland tumors are relatively uncommon lesions in clinical practice. Mucoepidermoid carcinomas and pleomorphic adenomas were the most common malignant and benign lesions, respectively. These tumors were more common in women aged over 40 years; the palate was the most common site.
